# Implementation and sustainability of hospital-based harm reduction delivery: a qualitative analysis

**DOI:** 10.1186/s12954-026-01476-0

**Published:** 2026-06-02

**Authors:** Leslie W. Suen, Leah Fraimow-Wong, Taylor Baisey, Kelly R. Knight, Laura Thomas, Ro Giuliano, Oanh Kieu Nguyen, Marlene Martín

**Affiliations:** 1 Division of General Internal Medicine, Department of Medicine, San Francisco General Hospital, University of California San Francisco, 2540 23rd Street, Floor 4, Room 4316, San Francisco, CA 94143 USA; 2 Division of Hospital Medicine, Department of Medicine, San Francisco General Hospital, University of California San Francisco, San Francisco, CA USA; 3 Department of Humanities and Social Sciences, University of California San Francisco, San Francisco, CA USA; 4 San Francisco AIDS Foundation, San Francisco, CA USA

**Keywords:** Harm reduction services, In-patient, Safety-net hospital, Qualitative study, Semi-structured interviews, Thematic analysis

## Abstract

**Background:**

Patients with substance use disorders (SUD) face several challenges when hospitalized, including stigma, undertreated withdrawal and pain, and higher rates of self-directed discharge. Hospital-based harm reduction services can support patients with SUD, increase engagement, build trust, and catalyze culture change. Our hospital’s Addiction Consult Team implemented a harm reduction program to provide education and substance use equipment to hospitalized patients whose SUD goals did not include abstinence. We conducted a qualitative evaluation of our harm reduction program to identify implementation and sustainability themes for long-term success.

**Methods:**

This qualitative study consisted of semi-structured interviews conducted between October 2022 and March 2023 with hospitalized patients receiving harm reduction services and hospital staff at an urban safety-net hospital in San Francisco, California. Interviews were audio-recorded and transcribed with informed participant consent. Transcripts were deductively and inductively coded through an iterative process, and we used thematic analysis to identify themes.

**Results:**

We interviewed 20 patients and 20 staff participants with interviews ranging from 30–45 min. We identified three themes related to implementation and three related to sustainability. Implementation themes were: (1) Operational barriers, including time constraints, lack of clearly defined roles, and competing demands, challenged delivery of harm reduction kits; (2) Tension between generalized versus specialized harm reduction program ownership limited impact; and (3) Educational gaps impeded broader harm reduction uptake and reinforced stigma. Sustainability themes were: (1) Experiential learning reinforced harm reduction practice and culture change; (2) Interprofessional collaboration, especially involving SUD champions, supported the program; and (3) Sharing patient success stories helped overcome stigma and burnout and highlighted harm reduction effectiveness.

**Conclusion:**

Harm reduction program implementation was challenged by resource constraints, complexity of program ownership, and lack of SUD knowledge. In contrast, experiential learning, interprofessional collaboration, and sharing patient successes sustained harm reduction efforts. Based on these findings, we recommend the following for long-term harm reduction program success: operationalize harm reduction workflow via hospital-wide interprofessional efforts; incorporate continuous, multimodal substance use training with all staff to ensure a baseline knowledge and drive culture change; and create feedback processes to share patient outcomes and narratives with staff.

## Background

One in nine hospitalized patients in the U.S. has a substance use disorder (SUD).^1^ While hospitals can be critical touchpoints for people with SUD, there are significant gaps in appropriately caring for this population during hospitalization.^2^ Hospitalized people with SUD experience stigma from staff, including viewing patients as violent and manipulative, which results in denying or delaying pain medication and shorter encounters with this patient population.^3,4^ Hospitals and healthcare workers often approach SUD care with a focus on abstinence, which can alienate patients, reduces engagement, and worsens health outcomes (including compromised patient-healthcare worker relationships,^5,6^ increased self-directed discharges,^7^ and incomplete treatment).^5,8^

Hospital-based harm reduction services are a powerful tool to improve outcomes for hospitalized patients with SUDs. The National Harm Reduction Coalition defines harm reduction as “a set of practical strategies and ideas aimed at reducing negative consequences associated with drug use” that incorporates social justice and human rights.^9^ Harm reduction in the community, such as needle exchange programs and naloxone distribution, has helped patients with SUD reduce substance use,^10^ increased linkage to substance use treatment,^10–12^ and decreased fatal overdoses.^13^ Expanding harm reduction to hospital settings is gaining momentum and has been found to increase patient engagement and trust,^14^ increase uptake of overdose prevention services,^15^ increase access to harm reduction supplies among marginalized populations,^14,16^ and catalyze culture change, including reducing stigma for patients with SUD.^14,17^ One program in Portland, Oregon, developed a nurse-led hospital harm reduction intervention embedded in its addiction consult team, leveraging nurses’ central role in hospital care to normalize harm reduction within hospital settings.^18^ Another program in Boston, Massachusetts, integrated a community-based harm reduction program into the local safety-net hospital via a formal “in-reach collaboration”, where community harm reduction specialists worked alongside the hospital’s addiction consult team to provide harm reduction supplies to hospitalized patients.^17^ Several hospitals, including emergency departments across the country, have integrated take-home naloxone distribution for patients at risk of overdose.^15,16^

In August 2020, our safety-net hospital’s interprofessional Addiction Consult Team (ACT) launched a harm reduction program to provide education and substance use equipment to hospitalized people not seeking substance use abstinence.^14,19,20^ ACT members performed a needs assessment among hospitalized patients with SUD to determine if harm reduction supplies were needed and what education and supplies would be most helpful.^18^ The supplies are provided as harm reduction kits and include needles, syringes, pipes, fentanyl test strips, and foil. ACT members, most commonly substance use navigators, provide these kits to patients, tailoring them to individuals’ needs, and provide them alongside counseling and education at discharge. Our prior work describes the program’s launch, beneficial impacts, and challenges faced by hospital staff and patients.^14,19^

While studies have described the development and initial outcomes of hospital-based harm reduction programs, none to our knowledge have examined program implementation and sustainability for long-term success. Thus, we conducted a qualitative study of our hospital’s harm reduction intervention to understand this knowledge gap, in hopes of improving care for hospitalized people with SUD.

## Methods

The study followed the Consolidated Criteria for Reporting Qualitative Research (COREQ). We previously described methods for this qualitative study.^14^ For this manuscript, we focused our analysis to answer the research question, “What are implementation and sustainability themes for effectively delivering harm reduction in our hospital long-term?” Briefly, we recruited participants from a single urban, academic safety-net hospital in San Francisco, California with an ACT where more than 80% of hospitalized patients are publicly insured and over a quarter (28%) have a SUD.^20,21^ All patients with a SUD seen by our ACT who do not seek abstinence are offered harm reduction interventions, but some decline. We recruited patients during hospitalization who were offered and received harm reduction interventions. We also recruited hospital staff who cared for those patients, including nurses (from the emergency department, labor and delivery, and medical-surgical units), attending and resident physicians, nurse practitioners, and ACT members. Given the prevalence of SUD in our hospital, all hospital staff across roles and departments could care for patients with a SUD. We used purposive sampling to include varied roles, disciplines, and views on harm reduction for staff, and varied languages, substances used, and racial and ethnic backgrounds for patients. We used semi-structured interview guides and demographic surveys to capture self-reported race, ethnicity, substances used, language, and staff’s hospital role. Two study members (LWS, LFW) conducted in-person or video conferencing software (Zoom Video Communications) interviews (approximately 30–45 min) between October 2022 and March 2023. All participants provided informed consent and received $50 for participation.

All interviews were audio-recorded, transcribed, and coded using Dedoose (version 9.0.85, Sociocultural Research Consultants). We developed a preliminary codebook using the interview guides, recordings, and memos. LFW and LWS coded interviews, and the codebook was iteratively refined during the coding process, with the addition of inductive codes drawn from the data. We performed thematic analysis with study members meeting regularly to review preliminary findings, discuss themes, and reconcile differences until consensus was met. The study was approved by the University of California, San Francisco Institutional Review Board.

## Results

Demographics of our participants have been previously presented.^14^ We interviewed 20 patients (mean age, 43 years; SD, 13) and 20 staff (mean age, 37 years; SD, 8). Participants identified as Black (n = 6, 30%), Latine (n = 6, 30%), White (n = 6, 30%), American Indian or Alaska Native (n = 1, 5%), and Asian and Pacific Islander (n = 1, 5%). Primary SUDs included co-occurring opioid and stimulant use (n = 7, 35%), stimulant use only (n = 7, 35%), opioid use only (n = 3, 15%), and alcohol use only (n = 3, 15%). We interviewed 8 (40%) nurses, 5 (25%) clinicians (including physician attendings and nurse practitioners), 4 (20%) physician trainees (including residents and fellows), and 3 (15%) substance use navigators and licensed vocational nurses. Some staff (n = 8, 40%) were ACT members, while the remaining worked in non-SUD roles in the hospital. We identified three themes related to implementation and three related to sustainability.

### Implementation themes

#### Theme 1: operational barriers, including time constraints, lack of clearly defined roles, and competing demands, challenged delivery of harm reduction kits

Staff cited how constraints on time, lack of clearly defined roles, and competing demands undermined staff’s ability to deliver harm reduction services consistently. Even when staff expressed personal commitment to harm reduction, the pressures of a busy hospital made harm reduction feel like a luxury they couldn’t afford: “*I want to do the right thing, but I also have four discharges and a new admit. It’s hard to do it all*.” (Nurse, Staff 6).

Staff struggled to integrate harm reduction amidst competing responsibilities and unclear workflows. Kits were not always easily accessible, their location in the hospital sometimes changed, and there was limited clarity on who was responsible for initiating or completing the distribution. Frontline nurses noted the challenge of adding new steps to an already complex and time-constrained discharge process. One floor nurse noted: *“I didn’t know where the supplies were or who was supposed to hand them out—by the time I figured it out, the patient was already gone.”* (Nurse, Staff 7).

Staff noted that supplies were most forgotten when a patient left early, was discharged overnight, or there was high turnover on the team. This affected patients, with one sharing, *“They said they were going to give me Narcan, but I never got anything. I think it got forgotten.”* (Patient 4) These logistical breakdowns were indicators of system-level design challenges. Staff frequently suggested the need for automation, such as standardized discharge ordersets in the electronic health record or alerts, and better integration into electronic health records to ensure patients consistently received supplies.

#### Theme 2: tension between generalized versus specialized harm reduction program ownership limited impact

Another challenge was the tension between normalizing harm reduction as a routine part of all staff roles versus maintaining it as a specialized task managed by the ACT. Staff expressed uncertainty about when and how they should initiate harm reduction conversations or distribute supplies, especially when an ACT consult had not been placed. One clinician shared, “*Sometimes I don’t know if I should be the one doing it or if ACT should handle it. It’s like, do I start the conversation or wait for the consult*?” (Resident, Staff 5) As another clinician described, “*Having a consult team is great, but it also makes it easy to think it’s not our job*.” (Nurse Practitioner, Staff 4) Without ACT, harm reduction tasks were often deprioritized. Patients noticed staff’s desire to help, though they lacked appropriate resources: “*They wanted to help, but there just weren’t enough people*.” (Patient 10).

While some participants felt that expanding responsibility across roles would increase sustainability, others worried that without adequate training and institutional support, this model could reduce harm reduction quality and consistency. Patients mirrored this tension. Some appreciated being seen by a specialized addiction team, while others wanted more engagement from their primary hospital teams: “*I liked the addiction team, but I also wanted my regular doctors to understand my using.*” (Patient 8).

#### Theme 3: educational gaps impeded broader harm reduction uptake and reinforced stigma

Staff described an urgent need for training in SUDs and harm reduction. The teaching hospital setting—with rotating residents, short-term learners, and evolving guidelines—meant that institutional knowledge dissipated quickly. As one resident clinician described, “*In med school we were told harm reduction was good, but we never actually learned how to do it.”* (Resident, Staff 5) Long-term staff also reported SUD knowledge gaps, particularly regarding harm reduction philosophy, tools, and language. In turn, patients noted when providers were unfamiliar with harm reduction supplies or unsure how to discuss substance use. One patient shared: “*Some nurses didn’t know what the test strips were. They had to ask someone else*.” (Patient 12).

Staff highlighted wanting to learn more about harm reduction and how it could help their patients. They stated that most of their exposure to harm reduction had been informed by stigmatizing media narratives, and learning the evidence could help address this stigma. One nurse recalled, *"People have a very skewed media-like view, where they’re like, 'Oh, why are we giving people pipes and needles?' But they don’t understand the disease behind it."* (Nurse, Staff 16).

### Sustainability themes

#### Theme 1: experiential learning reinforced harm reduction practice and culture change

While formal SUD training was lacking, as noted above, staff described a wide variety of informal or experiential learning opportunities that enabled sustainability. These included SUD and harm reduction talks at nursing huddles, bedside role modeling, and direct observation of ACT members. These micro-learning moments were often cited as the most practical and lasting. As one resident clinician described, “*We had a quick chalk talk on harm reduction… first time I actually learned how people use*.” (Resident, Staff 5).

Importantly, not all education was equally valued. Bedside teaching and role modeling left a deeper impression because it modeled concrete phrases for staff to use and allowed them to practice their skills firsthand: “*It helped when someone sat with me and showed me how to talk to the patient—not just [look at] slides*.” (Nurse, Staff 7) Staff especially appreciated learning from ACT: “*Honestly, I learn more from shadowing ACT than I did in training modules.*” (Nurse, Staff 8). Staff emphasized the importance of integrating harm reduction into the rhythm of clinical care, rather than treating it as a separate topic.

Patients, too, noticed when staff felt more comfortable engaging in harm reduction conversations. Encounters with nonjudgmental staff increased trust and sparked willingness to engage in care or consider behavior change, with one patient sharing: “*She didn’t judge me. I felt like she had been through it, too*.” (Patient 11) These interpersonal dynamics played a central role in shifting norms and attitudes across the institution.

#### Theme 2: interprofessional collaboration, especially involving SUD champions, supported the program

Effective interprofessional collaboration was key to successful harm reduction implementation. Staff noted that care improved when nurses, clinicians, social workers, and ACT members coordinated proactively. This teamwork bridged logistical gaps, ensured continuity, and fostered a shared sense of ownership. Patients also appreciated when their care felt cohesive and non-duplicative, with one patient noting, “*They all talked to each other — social worker, doctor, nurse. I felt like they were a team.*” (Patient 2).

ACT substance use navigators played a significant role in supporting staff and facilitating the delivery of harm reduction supplies. When navigators were available to teach staff about harm reduction supplies, conduct bedside education, and deliver supplies to bedside nurses, staff felt supported and more likely to engage. One participant noted, “*You [need the] navigators. Otherwise, it’s hard to implement*.” (Nurse Practitioner, Staff 4) Staff also felt that ACT made the work feel safer and more achievable, with one sharing, *"The Addiction Team really builds rapport with patients to see where they’re at… That’s something I never saw at other hospitals without a harm reduction approach."* (Nurse Practitioner, Staff 19).

Although ACT was often integral to harm reduction efforts, successful implementation also relied on informal non-ACT champions, such as knowledgeable nurses or attending physicians with the skillset to explain supplies, offer counseling, and troubleshoot issues in real time. This was especially useful since not all patients engage with ACT: “*Sometimes we encounter patients who are willing to engage with us as the primary team… but when you offer them ACT, they decline*.” (Nurse Practitioner, Staff 4).

#### Theme 3: sharing patient success stories helped overcome stigma and burnout and highlighted harm reduction effectiveness

Seeing and hearing real patient outcomes—such as reduced use, engagement in treatment, or improved stability—was a powerful motivator for staff. These stories circulated informally and helped overcome skepticism, counter moral fatigue, and reinforce that harm reduction was not only compassionate but also effective. As one nurse put it, “*Success stories help win over skeptical colleagues*.” (Nurse, Staff 7) Exposure to these stories made staff more receptive to harm reduction. These narratives were more persuasive than data or training and highlighted that harm reduction was about more than just offering supplies but also about addressing larger social determinants of health and whole-person care.

Staff reported that engaging in harm reduction improved their sense of purpose. They contrasted their current experience with prior hospitals where addiction was met with judgment or neglect: “*It feels good to work in a place where people aren’t judged for using*.” (Nurse, Staff 8) Patients echoed this sentiment, describing care that felt dignified and respectful. In some cases, this contrast alone was enough to re-engage patients with the healthcare system, as one participant shared: *“I didn’t get treated like a junkie here. That’s new.”* (Patient 14) Being able to offer harm reduction services aligned with staff’s values and made challenging work feel more meaningful. An ACT member reflected: “*If your role is about connection, not control, it’s more sustainable*.” (ACT Physician, Staff 9) By highlighting how harm reduction was an evidence-based action to improve patient care, the program was able to increase staff buy-in.

## Discussion

This qualitative study of a hospital-based harm reduction program identified six themes related to implementation and sustainability of our hospital-based harm reduction program. Our findings demonstrate how resource constraints, complexity of program ownership, and educational gaps can undermine harm reduction, but also how interprofessional collaboration, addiction education, and a nonjudgmental culture can sustain and scale harm reduction interventions over time. Our findings extend the growing literature on hospital-based harm reduction by highlighting the organizational learning processes that unfold beyond initial program development and implementation.

We identified several challenges to implementing hospital-based harm reduction that could be addressed through workflow operationalization to enhance the consistency of care.^22,23^ This finding is consistent with a systematic review that found that clear accountability of roles and responsibilities and embedding interventions into routine workflows were key facilitators to hospital-based interventions.^24^ The challenges of meeting individual patients’ needs highlighted in our study speak to well-documented challenges of providing care and enacting institutional change in resource-limited safety-net hospitals.^25,26^ Even when clinical teams were aligned in principle with harm reduction, the constraints of volume and urgent needs limited their ability to prioritize these interventions. These findings reinforce the necessity of institutional support, including time and dedicated personnel (e.g., navigators or harm reduction liaisons), for sustained program delivery.

Second, we identified tension between generalization and specialization of harm reduction responsibilities in our hospital with an addiction consult service. While some staff felt everyone should be equipped to engage in harm reduction, others viewed it as the purview of addiction specialists. Diffusing harm reduction responsibilities across all staff may reduce stigma and catalyze culture change,^14^ especially when leveraging interdisciplinary care teams.^27^ However, it introduces questions about the quality and fidelity of harm reduction interventions, risks misinformation, and may be unfeasible in resource-constrained environments. Furthermore, harm reduction practices can vary regionally and over time given the changing drug supply. Limiting harm reduction responsibilities to addiction specialists may be more feasible, reduce broader workforce burnout, and maintain harm reduction quality but limit integration, patient accessibility, and systemic culture change. Additionally, specialization of responsibilities may only be possible for hospitals with dedicated addiction consult services. Hospitals should balance resource allocation and specialized expertise, while ensuring interventions remain accessible and high-quality, by establishing clear workflows informed by multidisciplinary partners, systems-wide workflow operationalization,^24,28^ and baseline SUD education with staff.^14,29^

Our results underscore the need and desire for continuous SUD and harm reduction education for healthcare workers. Although our study sample benefitted from informal education, studies show a need for formal addiction education among healthcare workers.^30–32^ Educational interventions can reduce stigma and improve clinical outcomes for patients with SUDs,^22,33–35^ and in our study, staff education filled knowledge gaps and helped shift attitudes towards people with SUDs, leading to more compassionate patient care. Expanding education on harm reduction and SUDs across the health professions is essential for ensuring that harm reduction principles are integrated into standard healthcare practice.

Based on these findings, we offer three key recommendations to support the expansion and sustainability of hospital-based harm reduction (see Table 1). First, we recommend operationalizing harm reduction workflows via hospital-wide interprofessional efforts, such as creating harm reduction kit delivery ordersets with ownership assigned to ACTs or SUD champions, depending on resources. Second, we recommend incorporating continuous, multimodal substance use training with all staff to ensure hospital-wide baseline knowledge and drive culture change. Training could be experiential (e.g., bedside shadowing) and formal (e.g., lectures, onboarding training) to fill the gap in hospital-wide SUD and harm reduction education. Lastly, we recommend creating formal feedback processes to share patient outcomes and narratives with staff to help overcome stigma, resistance, and burnout.

**Table 1:** Recommendations to address harm reduction implementation and sustainability themes for long-term success in hospital setting

Recommendation	Theme addressed	Examples
Operationalize harm reduction workflow via hospital-wide interprofessional efforts	Operational barriers including time constraints, lack of clearly defined roles, and competing demands, challenged delivery of harm reduction kits	• Coordinate efforts and collaboration among key partners to allow maximal reach of patients while acknowledging competing staff demands, especially in hospitals without specialized addiction staff, to ensure sustainability • Assign ownership of education and supply delivery to specialized staff, such as substance use navigators, when possible, to ensure consistent delivery and education • If dedicated addiction staff are not available, assign to a non-addiction staff champion • Integrate harm reduction workflow into standard discharge protocols with clearly delineated responsibilities • Operationalize workflow through electronic health record prompts and ordersets • Create a quality assurance process to ensure patients receive supplies and that there is continuous improvement
	Tension between generalized versus specialized harm reduction program ownership limited impact	
	Interprofessional collaboration, especially involving SUD champions, supported the program	
Incorporate continuous, multimodal substance use training with all staff to ensure a baseline knowledge and drive culture change	Educational gaps impeded broader harm reduction uptake and reinforced stigma	• Establish ongoing harm reduction and substance use education efforts focusing on practical skills, local resources, and stigma reduction to meet staff needs and roles. These include: Experiential learning, such as bedside shadowing, nursing huddles, white board talks Formal education, such as departmental meetings, grand rounds, noon conferences, onboarding training • Leverage role modeling by establishing on-site harm reduction or addiction champions across disciplines who can model best practices, mentor peers, and lead educational efforts
	Experiential learning reinforced harm reduction practice and culture change	
Create formal feedback processes to share patient outcomes and narratives with staff	Sharing patient success stories helped overcome stigma and burnout and highlighted harm reduction effectiveness	• Use narrative medicine, debriefing, and case reflections to build healthcare worker buy-in and sustain morale • Create spaces and processes to share care transitions outcomes (e.g., linkages to primary care, connection to opioid treatment programs, residential intake) • Highlight individual and deidentified patient stories with care teams

Our study had several limitations, including being limited to the perspectives of patients that received harm reduction services and staff from a single hospital, limiting generalizability to other hospitals. Our findings must be interpreted within the context of our hospital setting. Harm reduction policies and regulations may also differ in other jurisdictions, and clinicians should refer to their local and state laws and regulations regarding the distribution of harm reduction supplies. Future research should explore multi-site harm reduction implementation across hospital contexts, including non-academic or rural settings, and evaluate the impacts and cost-effectiveness of different staffing and workflow models. Quantitative studies could assess the effect of harm reduction interventions on post-discharge outcomes, such as overdose, retention in care, or readmission. Importantly, future work must center people who use drugs not only as patients, but also as experts, leaders, and co-designers of hospital-based programs.

## Conclusions

This study highlights both the structural barriers and cultural shifts involved in implementing hospital-based harm reduction within a safety-net setting. While challenges related to logistics, staffing, and education persist, key strategies, such as interprofessional collaboration, role modeling, and centering patient success, enabled long-term program sustainability. Our recommendations center on operationalizing workflows, continuously educating all staff, and sharing patient experiences with staff. Embedding harm reduction into hospital culture not only improves care for people who use drugs but also enhances provider fulfillment and institutional resilience.

## Data Availability

The datasets generated and/or analyzed during the current study are not publicly available to maintain the anonymity of study subjects but are available from the corresponding author on reasonable request.

## Abbreviations

ACT: Addiction Consult Team
SUD: Substance Use Disorder

## References

[1] Suen LW, Makam AN, Snyder HR, et al. National prevalence of alcohol and other substance use disorders among emergency department visits and hospitalizations: NHAMCS 2014–2018. J Gen Intern Med. 2021;37(10):2420–8. 10.1007/s11606-021-07069-w. 34518978 10.1007/s11606-021-07069-w PMC8436853

[2] Englander H, Davis CS. Hospital standards of care for people with substance use disorder. N Engl J Med. 2022;387(8):672–5. 10.1056/NEJMp2204687. 35984354 10.1056/NEJMp2204687

[3] van Boekel LC, Brouwers EPM, van Weeghel J, Garretsen HFL. Stigma among health professionals towards patients with substance use disorders and its consequences for healthcare delivery: systematic review. Drug Alcohol Depend. 2013;131(1):23–35. 10.1016/j.drugalcdep.2013.02.018. 23490450 10.1016/j.drugalcdep.2013.02.018

[4] Peckover S, Chidlaw RG. Too frightened to care? Accounts by district nurses working with clients who misuse substances. Health Soc Care Community. 2007;15(3):238–45. 10.1111/j.1365-2524.2006.00683.x. 17444987 10.1111/j.1365-2524.2006.00683.x

[5] McNeil R, Small W, Wood E, Kerr T. Hospitals as a ‘risk environment’: an ethno-epidemiological study of voluntary and involuntary discharge from hospital against medical advice among people who inject drugs. Soc Sci Med. 2014;105:59–66. 10.1016/j.socscimed.2014.01.010. 24508718 10.1016/j.socscimed.2014.01.010 PMC3951660

[6] Curtis J, Harrison L. Beneath the surface: collaboration in alcohol and other drug treatment. An analysis using Foucault’s three modes of objectification. J Adv Nurs. 2001;34(6):737–44. 10.1046/j.1365-2648.2001.01803.x. 11422543 10.1046/j.1365-2648.2001.01803.x

[7] Simon R, Snow R, Wakeman S. Understanding why patients with substance use disorders leave the hospital against medical advice: a qualitative study. Subst Abus. 2020;41(4):519–25. 10.1080/08897077.2019.1671942. 31638862 10.1080/08897077.2019.1671942

[8] Brener L, Hippel WV, Kippax S, Preacher KJ. The role of physician and nurse attitudes in the health care of injecting drug users. Subst Use Misuse. 2010;45(7–8):1007–18. 10.3109/10826081003659543. 20441447 10.3109/10826081003659543

[9] National Harm Reduction Coalition. Principles of Harm Reduction. National Harm Reduction Coalition. 2024. Accessed April 16, 2024. https://harmreduction.org/about-us/principles-of-harm-reduction/

[10] Hagan H, McGough JP, Thiede H, Hopkins S, Duchin J, Alexander ER. Reduced injection frequency and increased entry and retention in drug treatment associated with needle-exchange participation in Seattle drug injectors. J Subst Abuse Treat. 2000;19(3):247–52. 10.1016/S0740-5472(00)00104-5. 11027894 10.1016/s0740-5472(00)00104-5

[11] Latkin CA, Davey MA, Hua W. Needle exchange program utilization and entry into drug user treatment: is there a long-term connection in Baltimore, Maryland? Subst Use Misuse. 2006;41(14):1991–2001. 10.1080/10826080601026027. 17162601 10.1080/10826080601026027

[12] Surratt HL, Otachi JK, Williams T, Gulley J, Lockard AS, Rains R. Motivation to change and treatment participation among syringe service program utilizers in rural Kentucky. J Rural Health. 2020;36(2):224–33. 10.1111/jrh.12388. 31415716 10.1111/jrh.12388 PMC7021582

[13] Smart R, Pardo B, Davis CS. Systematic review of the emerging literature on the effectiveness of naloxone access laws in the United States. Addiction. 2021;116(1):6–17. 10.1111/add.15163. 32533570 10.1111/add.15163 PMC8051142

[14] Fraimow-Wong L, Martín M, Thomas L, et al. Patient and staff perspectives on the impacts and challenges of hospital-based harm reduction. JAMA Netw Open. 2024;7(2):e240229. 10.1001/jamanetworkopen.2024.0229. 38386317 10.1001/jamanetworkopen.2024.0229 PMC10884877

[15] Riazi F, Toribio W, Irani E, et al. Community case study of naloxone distribution by hospital-based harm reduction program for people who use drugs in New York City. Front Sociol. 2021;6:619683. 10.3389/fsoc.2021.619683. 34307540 10.3389/fsoc.2021.619683 PMC8292929

[16] Sindhwani MK, Friedman A, O’Donnell M, Stader D, Weiner SG. Naloxone distribution programs in the emergency department: a scoping review of the literature. J Am Coll Emerg Physicians Open. 2024;5(3):e13180. 10.1002/emp2.13180. 38726467 10.1002/emp2.13180 PMC11079430

[17] Khan GK, Harvey L, Johnson S, et al. Integration of a community-based harm reduction program into a safety net hospital: a qualitative study. Harm Reduct J. 2022;19(1):35. 10.1186/s12954-022-00622-8. 35414072 10.1186/s12954-022-00622-8 PMC9002225

[18] Goff A, Lujan-Bear S, Titus H, Englander H. Integrating hospital-based harm reduction care—harnessing the nursing model. Substance Use &amp; Addiction Journal. 2024;45(2):307–13. 10.1177/29767342231219577. 38258867 10.1177/29767342231219577

[19] Perera R, Stephan L, Appa A, et al. Meeting people where they are: implementing hospital-based substance use harm reduction. Harm Reduct J. 2022;19(1):14. 10.1186/s12954-022-00594-9. 35139877 10.1186/s12954-022-00594-9 PMC8826677

[20] Martin M, Snyder HR, Coffa D, et al. Time to ACT: launching an Addiction Care Team (ACT) in an urban safety-net health system. BMJ Open Qual. 2021;10(1):e001111. 10.1136/bmjoq-2020-001111. 33500326 10.1136/bmjoq-2020-001111 PMC7843300

[21] Health Care Access and Information. Priscilla Chan & Mark Zuckerberg San Francisco General Hospital & Trauma Center. California Department of Health Care Access and Information. Accessed October 29, 2025. https://hcai.ca.gov/facility/priscilla-chan-mark-zuckerberg-san-francisco-general-hospital-trauma-center/

[22] Lindenfeld Z, Franz B, Cronin C, Chang JE. Hospital adoption of harm reduction and risk education strategies to address substance use disorders. Am J Drug Alcohol Abuse. 2023;0(0):1–10. 10.1080/00952990.2023.2169832. 10.1080/00952990.2023.2169832 36877147

[23] Jiao S, Bungay V, Jenkins E, Gagnon M. Opioid-specific harm reduction in the emergency department: how staff provide harm reduction and contextual factors that impact their capacity to engage in harm reduction practice. Harm Reduct J. 2024;21:171. 10.1186/s12954-024-01088-6. 39294704 10.1186/s12954-024-01088-6 PMC11409625

[24] Cowie J, Nicoll A, Dimova ED, Campbell P, Duncan EA. The barriers and facilitators influencing the sustainability of hospital-based interventions: a systematic review. BMC Health Serv Res. 2020;20(1):588. 10.1186/s12913-020-05434-9. 32594912 10.1186/s12913-020-05434-9 PMC7321537

[25] Chokshi Dave A, Cerise FP. Ethical issues in providing care in safety-net health systems. N Engl J Med. 2024;390(7):581–4. 10.1056/NEJMp2310893. 38345569 10.1056/NEJMp2310893

[26] Lindenfeld Z, Franz B, Lai AY, et al. Barriers and facilitators to establishing partnerships for substance use disorder care transitions between safety-net hospitals and community-based organizations. J Gen Intern Med. 2024;39(12):2150–9. 10.1007/s11606-024-08883-8. 38937366 10.1007/s11606-024-08883-8 PMC11347514

[27] Chang JE, Lindenfeld Z, Hagan H. Integrating harm reduction into medical care: lessons from three models. J Am Board Fam Med. 2023;36(3):449. 10.3122/jabfm.2022.220303R3. 37169587 10.3122/jabfm.2022.220303R3 PMC10636714

[28] Geerligs L, Rankin NM, Shepherd HL, Butow P. Hospital-based interventions: a systematic review of staff-reported barriers and facilitators to implementation processes. Implement Sci. 2018;13(1):36. 10.1186/s13012-018-0726-9. 29475440 10.1186/s13012-018-0726-9 PMC5824580

[29] Sharma M, Lamba W, Cauderella A, Guimond TH, Bayoumi AM. Harm reduction in hospitals. Harm Reduct J. 2017;14(1):32. 10.1186/s12954-017-0163-0. 28583121 10.1186/s12954-017-0163-0 PMC5460456

[30] von KlimoCampopiano M, Nolan L, Corbin M, et al. Physician reluctance to intervene in addiction: a systematic review. JAMA Netw Open. 2024;7(7):e2420837–e2420837. 10.1001/jamanetworkopen.2024.20837. 39018077 10.1001/jamanetworkopen.2024.20837 PMC11255913

[31] Bottner R, Bratberg J, Martin M, Weimer MB, Jordan A, Tierney M. Don’t “waive” goodbye to education for opioid use disorder. *NAM Perspectives*. Published online October 4, 2021. 10.31478/202110b 10.31478/202110b PMC8654468 34901777

[32] Horner G, Daddona J, Burke DJ, Cullinane J, Skeer M, Wurcel AG. “You’re kind of at war with yourself as a nurse”: perspectives of inpatient nurses on treating people who present with a comorbid opioid use disorder. PLoS ONE. 2019;14(10):e0224335. 10.1371/journal.pone.0224335. 31648259 10.1371/journal.pone.0224335 PMC6812769

[33] Crowther D, Curran J, Somerville M, et al. Harm reduction strategies in acute care for people who use alcohol and/or drugs: a scoping review. PLoS ONE. 2023;18(12):e0294804. 10.1371/journal.pone.0294804. 38100469 10.1371/journal.pone.0294804 PMC10723714

[34] Jakubowski A, Pappas A, Isaacsohn L, et al. Development and evaluation of a pilot overdose education and naloxone distribution program for hospitalized general medical patients. Subst Abus. 2019;40(1):61–5. 10.1080/08897077.2018.1518836. 30475162 10.1080/08897077.2018.1518836 PMC6778336

[35] Sell J, Visconti A. Harm reduction: assessing the educational needs of family medicine residents in care of persons who inject drugs. Fam Med. 2020;52(7):514–7. 10.22454/FamMed.2020.443447. 32640475 10.22454/FamMed.2020.443447

